# Psychometric properties and the prevalence, intensity and causes of oral impacts on daily performance (OIDP) in a population of older Tanzanians

**DOI:** 10.1186/1477-7525-4-56

**Published:** 2006-08-27

**Authors:** IA Kida, AN Åstrøm, GV Strand, JR Masalu, G Tsakos

**Affiliations:** 1 Centre for international health, UoB, Bergen, Norway; 2 Muhimbili University College of Health Sciences, Dar es Salaam, Tanzania; 3 Department of Odontology-Community Dentistry, UoB, Bergen, Norway; 4 Department of Odontology-Gerodontology, UoB, Bergen, Norway; 5 Department of Epidemiology and Public Health, University College of London Medical School, UK

## Abstract

**Background:**

The objective was to study whether a Kiswahili version of the OIDP (Oral Impacts on Daily Performance) inventory was valid and reliable for use in a population of older adults in urban and rural areas of Tanzania; and to assess the area specific prevalence, intensity and perceived causes of OIDP.

**Method:**

A cross-sectional survey was conducted in Pwani region and in Dar es Salaam in 2004/2005. A two-stage stratified cluster sample design was utilized. Information became available for 511 urban and 520 rural subjects (mean age 62.9 years) who were interviewed and participated in a full mouth clinical examination in their own homes.

**Results:**

The Kiswahili version of the weighted OIDP inventory preserved the overall concept of the original English version. Cronbach's alpha was 0.83 and 0.90 in urban and rural areas, respectively, and the OIDP inventory varied systematically in the expected direction with self-reported oral health measures. The respective prevalence of oral impacts was 51.2% and 62.1% in urban and rural areas. Problems with eating was the performance reported most frequently (42.5% in urban, 55.1% in rural) followed by cleaning teeth (18.2% in urban, 30.6% in rural). More than half of the urban and rural residents with impacts had very little, little and moderate impact intensity. The most frequently reported causes of impacts were toothache and loose teeth.

**Conclusion:**

The Kiswahili OIDP inventory had acceptable psychometric properties among non-institutionalized adults 50 years and above in Tanzania. The impacts affecting their performances were relatively common but not very severe.

## Background

Clinical data are mouth centered and rely on dental professionals' judgments. They have traditionally been utilized in assessing oral health in industrialized- and low income countries. Although informative, this clinical approach has been criticized because of its limited focus in terms of failing to consider functional and psychosocial aspects of oral health [1,2]. In response to a concern that clinical measures alone may not be adequate for assessing the public's oral health needs, oral health related quality of life measures (OHRQoL) have been developed and tested in various populations and are increasingly being used to supplement clinical indicators [1]. Cross-cultural adaptation of existing measures is warranted and efforts are ongoing to translate and adapt OHRQoL measures for use in non-western cultural settings [1,3].

One promising OHRQoL measure is the Oral Impacts on Daily Performance (OIDP) scale [4,5]. The OIDP was developed to measure oral impacts that seriously affect a person's daily life. It is based on the conceptual framework of the World Health Organisation's International Classification of Impairments, Disabilities and Handicaps, ICIDH [6], which has been amended for dentistry by LOCKER [7]. The OIDP concentrates only on the measurement of "ultimate" oral impacts, thus covering the fields of disability and handicap [4,5]. It consists of 8 items that assess the impact of oral conditions on basic activities and behaviours that cover the physical, psychological, and social dimensions of daily living [4,5]. Considering respondent burden, the OIDP is suitable for use in population surveys, not only in terms of being easier when measuring behaviours rather than feeling states, but also in being short. The scoring system quantifies (weigh) the impacts by using a score that reflects their frequency as well as a severity score that indicates the importance of the specific impact in the daily life of the person. Multiplying the frequency and severity scores provides different performance scores and the total score is expressed as a percentage of the sum of the performance scores divided by the maximum possible score multiplied by 100. In this sense the severity score provides a way of weighting the frequency of oral impacts with individually sensitive weights. Although, socio-dental indicators have been reported to perform satisfactorily as un-weighted rather than as weighted scores [8,9], the individually sensitive weighting system of the OIDP gives prominence and increased validity to the views of the respondents [10]. Moreover, it is evident that the OIDP weighted score is a better predictor than either the frequency or severity scores separately [1].

The OIDP has proved to be reliable and valid in cross-sectional population based studies. It has been shown to be applicable to older adult populations in Great Britain [11], Greece [10] and Thailand [12]. From Tanzania, Masalu et al [13] reported that the English OIDP frequency questionnaire fulfilled the psychometrical requirements underlying the scoring of the eight items and was applicable to adults attending higher education in Dar es Salaam.

Recently, it has been claimed that more oral health care is needed globally for the growing ageing populations [14]. In this context the OIDP index is worthy of consideration because of its adaptation for use in oral health needs assessment, thus making it useful for planning services [15,16]. This study aimed to assess the applicability of a Kiswahili version of the OIDP inventory for use in a population of older Tanzanian adults. First, internal reliability was assessed and discriminative and construct validity were determined by comparing OIDP scores of groups that differ regarding their demographic, socio-economic, clinical and behavioural characteristics. Secondly, the urban rural specific prevalence, severity and causes of oral impacts in older adults were assessed.

## Methods

### Study area

A cross sectional survey was conducted in Pwani region, Eastern Tanzania and in the capital city of Dar es Salaam from November 2004 to June 2005. According to the 2002 population and housing survey in Tanzania, Pwani region has the highest number of people 65 years and above in the country (7%). Dar es Salaam and Pwani region have a total population of 2.5 million and 889,154, respectively. The corresponding figures for population densities are 1,793 and 27 persons per square km. The districts have drinking water with fluoride content of about 1 mg fluoride/L (1 ppm)

### Sampling and procedure

**Figure 1:**
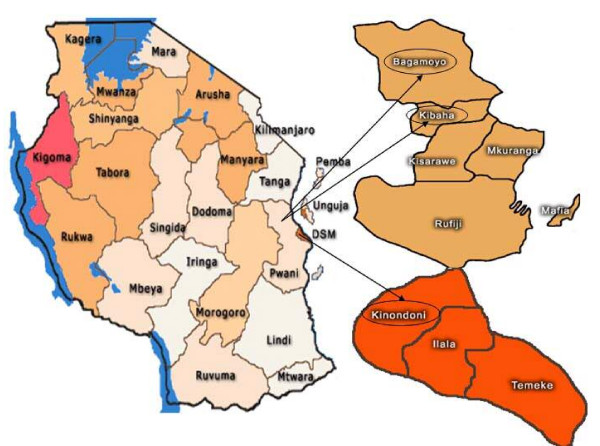
Tanzania: Kibaha and Bagamoyo districts (rural) in Pwani region and Kinondoni district (urban) in Dar es Salaam city.

A sample size of 1200 was calculated assuming a prevalence rate of tooth loss (≥ 1 missing tooth) of 50%, a precision of 4% and a design effect of 2 [17]. The estimated sample size was satisfactory also for two sided tests, assuming prevalence of oral impacts of 0.60 and 0.50 in individuals with caries experience and without caries experience, a significance level of 5% and a power of 90% [17]. A stratified (disproportionate) two-stage cluster sample design with villages as the primary sampling unit was implemented. Villages were selected from two rural districts (Kibaha and Bagamoyo) and one urban (Kinondoni) district in Pwani and Dar es Salaam region, respectively (Fig 1). To obtain a sample of older adults of mixed socio-economic background, 107 pure urban (N = 59688) villages and 96 pure rural villages (N = 26520) were listed in Kinondoni and in Kibaha/Bagamoyo. At the first stage, 10 pure urban villages (n = 6290) and 10 pure rural villages (n = 3729) were selected by systematic random sampling from the district village population lists. At the second stage, a total of 60 households were selected by systematic random sampling from each village selected at the first stage. This involved randomly selecting the first household by spinning a bottle at the presumed center of each village to obtain a starting direction, listing on papers all household heads in the selected direction up to the border of the village, folding the paper and randomly picking one name. The next household would be one whose front door was nearest to the previous one. A household was defined as a group of people living, cooking and eating together. One person 50 years and above was enrolled per household. In case the household had several people in the targeted age group, one man and one woman were selected randomly. Over sampling of rural villages were implemented to achieve a sample size that was big enough to conduct stratified analyses. A village leader followed the data collectors through the village and traditional village customs were observed to ensure a high response rate. Only consenting subjects were included in the study. Reasons for non-participation were refusals (n = 45), absence from the household on the day of the interview (n = 88). Exclusion criteria were presence of disease/conditions that might pose a health risk to the participant or that may interfere with the interview and clinical examination. Subjects were excluded if they were ill or had a history of psychiatric problems (n = 23), were intoxicated with alcohol (n = 2), were too old (n = 7) or had beliefs in witchcraft (n = 4). Permission to carry out the study was approved by the Research and Publication Committee at Muhimbili University College of Health Sciences, MUCHS, regional and district administration authorities, village leaders and from the ethical research committee in Norway (REK VEST). Informed consent was obtained from all participating subjects.

### Interview

For the OIDP inventory to be administered among older adults 50 years and above in Tanzania, translation into Swahili language was mandatory (see additional file 1). Kiswahili is the national and official language in Tanzania and almost all (95%) Tanzanians speak the language proficiently. A structured interview schedule, including the OIDP inventory, questions on socio-demographic characteristics and other health-and oral health issues was constructed in English, translated into Kiswahili by two Tanzanian professionals fluent in Kiswahili and English and back translated into English by two independent translators. Project staff at the MUCHS reviewed the OIDP questionnaire for semantic, experiential and conceptual equivalence to the source version. Sensitivity to culture and selection of appropriate words were considered. After being reviewed for content and face validity by panels of Tanzanian academics, the Kiswahili version of the OIDP inventory was compared with a *de novo *development of oral impacts on daily performances generated through focused group interviews with a sub-group of the study participants. The interview schedule was piloted before administration to identify questions which were not clear. The interview was administered in the field by two trained research assistants before the participants were clinically examined.

The eight item OIDP index referred to difficulty carrying out the eight daily life activities during the past six months, (Table 1). Each frequency item (originally scored 0–5) was changed into 0–3 scores where (0) never, (1) less than once a month, (2) once or twice a month up to once or twice a week, (3) 3–4 times a week or more often [18]. The OIDP severity scores were assessed on a 4-point scale as follows; (0) not severe at all, (1) less severe, (2) severe, (3) very severe. Finally the participants were asked to identify the oral condition that caused the specific impacts by answering for each reported item (1) yes or (0) no to the following alternatives: "toothache, loose teeth, gum abscess, bad breath and bleeding gums".

**Table 1:** The Oral Impacts on Daily Performances index (OIDP).

During the past 6 months – how often have problems with your mouth and teeth caused you any difficulty in:	
a.	Eating and enjoying food
b.	Speaking and pronouncing clearly
c.	Cleaning teeth
d.	Sleeping and relaxing
e.	Smiling, laughing and showing teeth without embarrassment
f.	Maintaining usual emotional state without being irritable
g.	Carrying out major work or socio role
h.	Enjoying contact with people

Performance scores representing the weighted impact on each performance were calculated by multiplying frequency (0–3) and severity scores (0–3). The overall OIDP impact scores, OIDP-total, was the sum of all 8 weighted performances (range 0–72). For the purpose of cross-tabulation and logistic regression analyses, the OIDP-total scores were dichotomized using a score of 1 or more as cut-off. The distribution of the OIDP-total scores supported this cut-off point. Following the alternative scoring method described by Gherunpong et al. [18], each weighted performance score (range 0–9) was classified into 6 levels of intensity; none, very little, little, moderate, severe and very severe (Table 2). The overall intensity of oral impacts for a person follows the same classification and refers to the most severe impact on any of the 8 performances or the highest performance score. Finally, the extent of oral impacts, OIDP-extent, (range 0–8) was calculated as a simple count score (OIDP SC); i.e. summing dichotomized frequency items in terms of (1) affected (including the original categories 1,2,3) and (0) not affected (including the original category 0). In order to demonstrate the relative burden of impacts among those affected, in this study we report on the intensity and extent of oral impacts among those participants with an impact, not on the whole sample. This means that for this purpose we do not consider subjects scored as zero respectively ("none" for intensity, "not affected" for extent), as this information is already provided by the prevalence figures. The correlation coefficient (Spearman's Rho) between the weighted OIDP-total on the one hand and OIDP SC sum scores on the other was 0.97.

**Table 2:** Classification of the intensity of oral impacts on a performance, after Gherunpong et al., 2004 [18].

Intensity	Severity score		Frequency score	Performance score
Very severe	(3)	×	(3)	9
Severe	(3)	×	(2)	6
	(2)	×	(3)	
Moderate	(2)	×	(2)	4
	(3)	×	(1)	3
	(1)	×	(3)	
Little	(2)	×	(1)	2
	(1)	×	(2)	
Very little	(1)	×	(1)	1
No impacts	(0)	×	(0)	0

The predictor variables used in the analyses, their coding and the number of subjects (%) according to categories are depicted in Table 3. *Socio-demographics *were assessed in terms of place of residence, gender and age. *Family wealth *was assessed as an indicator of socio-economic status in accordance with a standard approach in equity analyses [19]. Household durable assets indicative of family wealth (e.g. bicycle, television, car, motor cycle) assessed as (1) available/in working condition, (2) not available/available but not in working condition were analyzed in a principle component analysis. The first component resulting from the analysis was used to divide households into four approximate quartiles of wealth status ranging from 1^st ^quartile (least poor) to 4^th ^quartile (most poor). *Self reported oral health status *was coded (1) very good, (2) good, (3) average, (4) bad, (5) very bad and further dichotomized into (1) good (original categories 1,2.3) and (2) bad.

**Table 3:** Frequency distribution of participants in urban (Kinondoni) and rural (Kibaha/Bagamoyo) districts of Tanzania according to category on independent variables (n = 1031).

		Urban	Rural
Variables	Categories (Code)	% (n)	% (n)

*Age (years)*	50–59 (1)	50.3 (257)	37.9 (197)
	60–69 (2)	28.8 (147)	30.0 (156)
	70+ (3)	20.9 (107)	32.1 (167)*
*Gender*	Male (1)	42.7 (218)	50.0 (260)
	Female (2)	57.3 (293)	50.0 (260)*
*Wealth index*	1^st ^quartile-least poor	45.4 (232)	4.4 (23)
	2^nd ^quartile	40.1 (205)	8.8 (46)
	3^rd ^quartile	11.2 (57)	35.0 (182)
	4^th ^quartile – poorest	3.3 (17)	51.7 (269)*
*Number of decayed teeth*	0–1 (0)	54.0 (276)	44.6 (232)
	2–22 (1)	46.0 (235)	55.4 (288)*
*Posterior occluding units*,	10 POU (1)	12.1 (62)	22.7 (118)
	0–9 POU (2)	87.9 (449)	77.3 (402)*
*Mobile teeth*	0–1 (1)	83.8 (428)	77.3 (402)
	2–25 (2)	16.2 (83)	22.7 (118)*
*Self-reported oral health status*	Good (1)	74.4 (380)	54.4 (283)
	Bad (2)	25.6 (131)	45.6 (237)*
*Chewing ability*	All foods (1)	74.8 (382)	63.7 (331)
	Soft/mashed only (2)	25.2 (129)	36.3 (189)*
*Number of missing teeth*	0–10 (1)	83.2 (425)	82.2 (427)
	11–19 (2)	11.9 (61)	12.3 (64)
	20+ (3)	4.9 (25)	5.6 (29)

* *p *≤ 0.05.

The total number in the different categories did not add up to 1031 owing to missing values.

### Clinical examination

One trained and calibrated dentist (IK) conducted all clinical examinations in a shaded area with natural daylight as the source of illumination and with an assistant recording the observations. Research assistants for recording were trained and calibrated before the main survey. Participants identified with problems that needed treatment were referred or advised to seek treatment from the nearest health care facility. Oral health education sessions were provided for all the participating subjects. A full mouth clinical examination, including 3^rd ^molars was conducted. *Caries experience *was assessed in accordance with the criteria described by the World Health Organization, WHO [20]. *Number of teeth lost due to any reason *was calculated with the inclusion of edentulous people (0.6%) and coded (1) 0–10, (2) 11–19 and (3) 20+. *Tooth mobility *was assessed using a modified Miller's index [21], whereby the ends of two instruments were placed on either sides of the tooth and forces applied in bucco-lingual/palatal direction and scored as present or absent. An individual tooth mobility score was defined as (1) 2 or more mobile teeth, (0) less than 2 mobile teeth. *Posterior premolar and molar occluding units, POU*, were counted based on existing natural tooth contacts between maxilla and mandible in the bilateral regions. The number of occluding units, POU, (with or without intact anterior region) was categorized into (1) complete posterior occluding support/10 functional occluding units, (2) reduced posterior occluding support/1–9 occluding units and (3) absence of bilateral occluding support. For analysis, a dummy variable was constructed yielding, (1) reduced occluding support (0–9 units) and (0) complete occluding support (10 units). The distribution of the originally scored POU variable supported this cut off point.

### Reproducibility

Duplicate clinical examinations were carried out on a randomly selected sub-sample, considered to be representative of the study subjects. Analysis performed on the duplicate examination recordings gave kappa statistics of 1.00 for missing teeth due to caries, decayed teeth and occluding support. Kappa statistics of 0.77 and 0.79 were provided with respect to mobile teeth and tooth loss due to other reasons, respectively. These figures indicate a very good intra-examiner reliability according to WHO [20].

### Statistical analyses

Data were analyzed using SPSS version 13.0. Due to the very low number of edentulous subjects in the material (six subjects), edentate subjects were included in the analysis. Limiting the analyses to the dentate participants did not change the results reported here. Cross tabulation and chi-square statistics were used to assess bivariate relationships. Internal consistency reliability was assessed using Spearman's correlation coefficient and Cronbach's alpha. To adjust for the effect of the survey design (strata and clustering), re-analyses were conducted with STATA 9.0 using the svylogit command.

## Results

### Characteristics of participants

A total of 511 (participation rate 85.2%) urban and 520 (participation rate 86.7%) rural subjects between 50 and 100 years (mean age: 62.9, SD = 10.6, men: 46.4%, no formal education: 44.7%), completed an extensive personal interview followed by a full mouth clinical examination. The prevalence of tooth loss (≥ 1 tooth due to any reason) was 85.5% (mean tooth loss 6.1, SD = 6.4) in urban areas and 82.1% (mean tooth loss 5.9, SD = 6.6) in rural areas [22]. Table 3 gives the percentage distribution of participants' socio-demographic-, clinical-, and behavioral characteristics in urban Kinondoni and rural Kibaha/Bagamoyo districts.

### OIDP validity and reliability

One subject omitted one OIDP frequency item. This small number of missing responses adds support to the face validity of the Kiswahili OIDP inventory successfully addressed through focused group interviews and panel reviews. Construct and criterion validity was demonstrated in that the OIDP-total impact scores discriminated in the expected direction between subjects who rated their oral health status and chewing ability as good and bad (Table 4). Moreover, as depicted in Table 4, the mean OIDP total scores increased significantly with increasing number of decayed teeth, reduced number of posterior occluding units, increased number of mobile teeth (both urban and rural) and increased number of missing teeth (urban only). The association between the prevalence of oral impacts (OIDP total >0) and factors known to be associated with oral health; socio-demographic-, clinical and behavioral variables were assessed using unadjusted and adjusted logistic regression analysis (Table 5). There was a statistically significant relationship (p < 0.001) between the prevalence of oral impacts and place of residence, wealth index, self-reported oral health status, chewing ability and a number of clinical oral health indicators in the bivariate analysis. In the multiple logistic regression analysis, age, number of POU's, self-rated oral health and reported chewing ability remained statistically significant predictors. The ORs for experiencing any oral impact was 0.6, 1.7,7.7 and 3.2 if being older, having reduced number of POU's, reporting bad oral health status and reporting chewing problems, respectively.

**Table 4:** Construct and criterion validity of the OIDP-total scores: mean values for each category of grouping variable and differences in mean rank (DMR). Mann Whitney U test and Kruskal Wallis test.

	Urban (n = 508)			Rural (n = 512)		
	Mean	*p*	DMR	Mean	*p*	DMR

*Oral health status*						
Good	2.1			3.6		
Bad	8.9	0.001	142.3	15.6	0.001	167.2
*Chewing foods*						
All kinds	2.5			5.3		
Soft and mashed only	7.7	0.001	100.0	15.7	0.001	136.2
*Decayed teeth*						
0–1	2.9			7.5		
2–22	4.9	0.002	37.8	10.4	0.002	38.8
*Occluding units*						
10 units	1.4			6.4		
0–9 units	4.2	0.001	67.1	9.9	0.001	51.9
*Number of missing teeth*						
0–10	3.6			8.3		
11–19	4.6			13.3		
20+	6.2	0.492	26.1	11.0	0.001	59.8
*Mobile teeth*						
0–1	3.5			8.2		
2 or more	5.3	0.034	35.0	12.1	0.001	57.2

**Table 5:** Odds ratios (ORs) and 95% Confidence Limits (CL) for having any oral impacts on daily performance (OIDP total >0) according to clinical and non-clinical variables.

		Unadjusted		Adjusted	
	%(n)	OR	95%CI	OR	95% CL
*Age (years)*: 50–59	57.1 (257)	1		1	
60–69	50.7 (151)	0.8	0.5–1.1	**0.6**	**0.4–0.8**
70+	58.1 (158)	1.0	0.7–1.4	0.7	0.4–1.0
*Sex*: Male	54.8 (258)	1		1	
Female	56.1 (308)	1.0	0.8–1.3	0.9	0.6–1.2
*Place*: Urban	48.8 (248)	1		1	
Rural	62.1 (318)**	**1.6**	**1.2–2.1**	1.2	0.9–1.7
*Wealth*: 1^st ^least poor	50.2 (127)	1		1	
2^nd^	48.0 (120)	0.9	0.6–1.2	0.5	0.5–1.1
3^rd^	59.1 (139)	1.4	1.0–2.0	0.5	0.5–1.1
4^th ^most poor	63.8 (180)**	**1.8**	**1.4–2.4**	0.4	0.4–1.1
*OHS*: Good	38.0 (249)	1		1	
Bad	86.8 (317)**	**10.6**	**7.5–15.0**	**7.7**	**5.4–11.1**
*Chewing food*: all	44.3 (313)	1		1	
soft	80.6 (253)**	**4.9**	**3.6–6.7**	**3.2**	**2.1–4.7**
*Decayed teeth*: 0–1	49.5 (249)	1		1	
2–22	61.3 (317)**	**1.6**	**1.3–2.1**	0.9	0.7–1.3
*Missing teeth*: 0–10	53.4 (449)	1		1	
11–19	64.0 (80)	**1.5**	**1.0–2.1**	0.6	0.4–1.1
20+	68.5 (37)*	**1.8**	**1.0–3.2**	0.6	0.2–1.3
*Posterior Occl Units*:					
10	41.0 (73)	1		1	
0–9	58.6 (493)**	**2.0**	**1.4–2.8**	**1.7**	**1.2–2.6**
*Mobile teeth*: 0–1	52.1 (428)	1		1	
2 or more	69.3 (138)**	**2.0**	**1.4–2.8**	1.4	0.9–2.1

The total number in the different categories did not add up to 566 owing to missing values. ** p ≤ 0.001.

Test-retest reliability of the OIDP inventory was not performed due to ethical considerations, because oral health education sessions were provided for all participants after completion of the oral examination and because referrals for treatment were given to those with an acute oral problem. Internal consistency reliability analysis showed homogeneity of the OIDP-total items. In Kinondoni (urban), the corrected item – total correlation coefficient (i.e the correlation between each item and the total score after omitting the item ranged between Spearman's rho 0.42 and 0.64 with a standardized Cronbach's alpha coefficient of 0.83. In Kibaha/Bagamoyo (rural) the corrected item total ranged from Spearman's rho 0.62 to 0.82 with a Cronbach's alpha of 0.90 (Table 6).

**Table 6:** Internal consistency reliability of the Kiswahili version of the Oral Impacts on daily Performances (OIDP) inventory among urban and rural participants: Corrected item total Spearman's correlation and Cronbach's alpha if item deleted

OIDP item	Urban (n = 508)		Rural (n = 512)	
	Corrected item total correlation	Alpha if item deleted	Corrected item total correlation	Alpha if item deleted

1. Eating	.46	.81	.62	.91
2. Speaking	.54	.77	.70	.89
3. Cleaning	.42	.78	.63	.91
4. Sleeping	.64	.75	.77	.89
5. Showing teeth	.56	.76	.63	.90
6. Emotion	.64	.75	.82	.89
7. Work	.51	.77	.78	.89
8. Social contact	.59	.77	.79	.89

Standardised Cronbach's Alpha		0.83		0.90

### Prevalence, extent, intensity and causes of OIDP

A total of 43.2% and 44.5% had impact scores of zero (floor effect) using the OIDP ADD and the OIDP-total scoring method, respectively. The corresponding ceiling effects (proportions of adults who scored maximum) were 0.6% and 0.1%. As shown in Table 7 and 8, the prevalence of oral impacts (OIDP total >0) was high, amounting to 51.2% and 62.1% in Kinondoni (urban) and Kibaha/Bagamoyo (rural), respectively. In both areas, impacts on eating were most prevalent (42.5% in urban and 55.1% in rural) followed by cleaning teeth (18.2% in urban and 30.6% in rural), emotional stability (17.4% in urban and 30.4% in rural) and sleeping/relaxing (12.1% in urban and 27.0% in rural). Impacts on social contacts, work and smiling/showing teeth were the least prevalent impacts in both areas (Tables 7, 8). However, they were still quite prevalent; 5.9% of urban and 21.7% of rural participants reported oral impacts in relation to social contacts, while the figures for oral impacts in relation to smiling were 8.4% and 15.6%.

**Table 7:** Prevalence (% OIDP SC >0), mean OIDP total impact scores and intensity (% of adults with oral impacts) of older Tanzanians in urban areas (n = 508)

	Overall	Eating	Speaking	Cleaning	Sleeping	Smiling	Emotion	Work	Contact
	(n = 508)	(n = 511)	(n = 508)	(n = 511)	(n = 511)	(n = 511)	(n = 511)	(n = 511)	(n = 511)
OIDP prevalence %	51.2	42.5	9.1	18.2	12.1	8.4	17.4	7.6	5.9
									
OIDP impact score:									
*Range*	0–40	0–9	0–6	0–9	0–6	0–9	0–9	0–6	0–6
*Mean (sd)*	3.8 (6.5)	1.4 (2.1)	0.3 (1.0)	0.5 (1.4)	0.4 (1.1)	0.3 (1.3)	0.5 (1.3)	0.2 (0.9)	0.2 (0.8)
									
Impact intensity ^a^									
*Very little*	11.2	11.5	8.7	30.1	11.3	14.0	14.6	12.8	26.7
*Little*	22.0	31.8	43.5	29.0	19.4	25.6	38.2	30.8	26.7
*Moderate*	58.4	38.7	37.0	30.1	62.9	39.5	38.2	53.8	36.7
*Severe*	8.4	12.0	10.9	6.5	6.5	7.0	7.9	2.6	10.0
*Very severe*	0.0	6.0	0.0	4.3	0.0	14.0	1.1	0.0	0.0

^a ^Impact intensity:% of adults with impact.

**Table 8:** Prevalence (% OIDP SC >0), mean OIDP-total impact score-, and impact intensity scores (% of adults with impacts) of older Tanzanians in rural areas (n = 512)

	Overall	Eating	Speaking	Cleaning	Sleeping	Smiling	Emotion	Work	Contact
	(n = 512)	(n = 519)	(n = 514)	(n = 520)	(n = 519)	(n = 520)	(n = 520)	(n = 520)	(n = 520)
OIDP prevalence %	62.1	55.1	20.8	30.6	27.0	15.6	30.4	22.5	21.7
									
OIDP impact score:									
*Range*	0–72	0–9	0–9	0–9	0–9	0–9	0–9	0–9	0–9
*Mean (sd)*	9.1 (13.3)	2.1 (2.6)	0.9 (1.9)	1.3 (2.3)	1.1 (2.1)	0.6 (1.7)	1.2 (2.2)	0.9 (1.9)	0.9 (1.9)
									
Impact intensity^a^									
*Very little*	11.9	18.1	11.2	12.6	7.9	11.1	14.6	7.7	7.1
*Little*	15.8	18.4	12.1	13.8	17.1	13.6	19.6	21.4	21.2
*Moderate*	60.7	32.6	40.2	35.8	50.0	50.6	36.7	47.9	43.4
*Severe*	11.8	22.2	29.9	27.7	18.6	16.0	19.6	15.4	22.1
*Very severe*	0.0	8.7	6.5	10.1	6.4	8.6	9.5	7.7	6.2

^a ^Impact intensity:% of adults with impact.

In terms of the extent of oral impacts among subjects with impacts, in Kinondoni (urban) 47.3% had 1, 18.2% had 2 and 9.3% had 3 impacts. The corresponding figures in Kibaha/Bagamoyo (rural) were 32.7%, 13.0% and 11.4%. Few participants had 5 or more impacts.

In relation to the intensity of impacts, 6.0%, 14.0% and 4.3% of the participants in Kinondoni (urban) with impacts on respectively, eating, smiling and cleaning, had *very severe *impacts. Corresponding figures for eating, cleaning, emotion and smiling were 8.7%, 10.1%, 9.5% and 8.6% in Kibaha/Bagamoyo (rural). Mean scores of impacts (range 0–9) on each of the 8 performances ranged from 1.4 (eating) to 0.2 (working/social contact) in urban areas and from 2.1 (eating) to 0.6 (smiling) in rural areas. The distribution of the OIDP-total scores were skewed, mean 3.8 (sd = 6.5, range 0–40) and mean 9.1 (sd = 13.3, range 0–72) in urban and rural areas (Table 7, 8).

**Figure 2:**
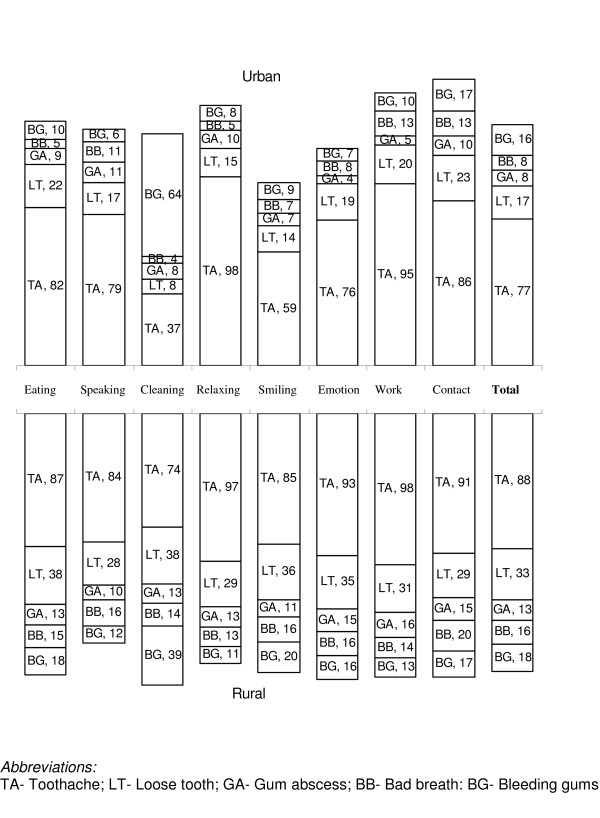
Percentage of the main oral problems causing an impact on the eight performances according to place of residence.

The oral problems perceived to cause the impacts on each of the 8 performances are shown separately for urban and rural residents in Fig. 2. In both areas, toothache and loose teeth were the most frequently perceived causes of impairments for almost all the performances. The majority of impacts on cleaning teeth were caused by bleeding gingiva and toothache in urban and rural areas, respectively. Bad breath was the third most frequently reported cause of impacts on speaking (among both urban and rural subjects) and enjoying contact with people (rural subjects), while bleeding gums was the third most frequently reported cause of impacts on enjoying contact with people in the urban areas.

## Discussion

The present study applied for the first time a Kiswahili version of the OIDP weighted inventory to a population of older adults in urban and rural cultural settings of Tanzania. This necessitated reestablishment of the psychometrical properties and a further evaluation of the validity of the OIDP scale. When used in personal interviews, the Kiswahili OIDP was valid and reliable with psychometric properties similar to the original English version [10,11] and to the English version shown to be applicable with Tanzanian students of higher education [13]. Internal consistency reliability in terms of Cronbach's alphas of 0.83 (urban) and 0.90 (rural) were satisfactory and well above the recommended levels of 0.70. Moreover, the corrected item-total correlation coefficients, ranging from Spearman's rho 0.42 to 0.64 in the urban area and from Spearman's rho 0.62 to 0.82 in the rural area, were above the minimum level of 0.20 for inclusion of an item into a scale [23]. Cultural issues, in particular language might give rise to problems with validity. Although no approach guarantees cross-cultural equivalence, the Kiswahili OIDP seemed to preserve the overall concepts of the English version and did not differ in terms of sequence of questions, the Likert scale and recall memory period used. Experience of the usability of the OIDP inventory across multicultural populations of Tanzania, first applied in English as a self-administered questionnaire [13] and recently in Kiswahili as personal interviews provided further support for the cross-cultural equivalence of this inventory.

Hypotheses regarding the construct and criterion validity of the Kiswahili OIDP inventory were confirmed in that the weighted scores varied systematically and in the expected direction with self-reported oral health status and perceived chewing ability (Table 4, 5). The validity of the Kiswahili translation is supported by observations similar to those in the UK [11], Thailand [4,5], Greece [10] Norway[24] and among university students in Dar es Salaam [13]. In addition, the OIDP scores were significantly associated with various clinical measures (Table 4). In a study of Greek adults 65 years and above, Tsakos et al [2] reported significant associations between various clinical indicators and OIDP, after adjusting for socio-demographic variables. An important finding of this study was the relationship with number of POUs, a clinical indicator reflecting both the number of posterior teeth present as well as their function. Similar results have been reported by Tsakos et al., [2], Srisilapanan and Sheiham [12], Locker and Slade [25], Gilbert et al. [26] and Sarita et al [27]. Clinical measures have traditionally been excluded from previous validations of the OIDP instrument [10,11]. The rationale behind the decision to omit clinical variables is derived from the conceptual distinction between health and disease [28,29]. Consistent with this reasoning and with findings reported previously [13], the self-rated oral health status and reported chewing ability associated more strongly with the OIDP impact score than did the measures of clinical indicators. According to the results depicted in Table 5, the ORs for having any oral impact was 7.7 if reporting bad oral health status, 3.3 if eating soft foods only and 1.7 if having reduced number of POUs.

As shown in Table 5, rural- and poor participants scored higher on the OIDP inventory than their urban- and less poor counterparts. Accordingly, Srisilapanan [12] found older Thai adults with a high income to be more likely to have low OIDP scores while their counterparts with low income tended to have high OIDP scores. This finding is similar to those observed with other indicators, showing that reducedOHRQoL is most commonly recorded in socially and economically disadvantaged groups [29]. An inverse relationship between OIDP and age emerged in multivariate analysis when allowing for the effects of other variables (Table 5). Similar results have been observed in Norway, with a different age classification system and might reflect the changes in expectations occurring with increasing age [30]. Recently, Locker and Gibson [31] found that half of the elderly subject investigated who described their oral health as poor, were still satisfied with their oral health status, a finding that was attributed to changes and adjustment of values and expectations in later life.

About one-half of the urban and rural subjects interviewed had experienced at least one oral impact during the past 6 months. The estimates obtained compares to the prevalence of impacts reported in Thai populations of younger (35–44 years) (73%) and older adults (52%) [5,12]. The present prevalence is higher, however, than that reported among older adults (67–79 years) in a national survey from Norway (18%) [24] as well as in Great Britain (12.3%) [11] and Greek (39.1%) [10] dentate older populations using the same socio-dental indicator. Further research is required to examine whether the differences in prevalence of OIDP between occidental and non-occidental societies are related to differences in dental status or in culture specific responses to dental impairments.

Consistent with previous studies and across age groups, eating was the most commonly reported aspect of OHRQoL [13,30,32]. The percentage of impacts related to eating observed among younger and older Tanzanian adults were similar to those observed in comparable age groups of younger and older Thais, but much higher than the impacts of dentate adults from Greece (29.9%), UK (7.0%) and Norway (11.3%) [10,11,24]. More than half of the urban and rural adults with impacts reported having very little, little and moderate intensity, indicating that despite their relatively high prevalence, the reported impacts were not severe. In urban adults, impacts in relation to smiling and showing teeth were more severe than impacts on other performances, whereas in rural adults cleaning was the most severe impact followed by emotional stability and eating. Consistent with results obtained among Thai adults [4,5], toothache and loose teeth were the most frequently reported reasons for impacts from eating.

It should be noted that the accuracy of reporting perceived impairments and symptoms in population based studies might be limited. Another caveat might be the OIDP inventory using a recall period of 6 months and relying on self-reports which implies it can be prone to recall bias. Compared to shorter recall periods longer recalls might result in an underestimation of health consequences but might provide valid estimates for severe outcomes [33]. This might be the case with the OIDP covering ultimate impacts thus essentially measuring the disabilities and handicaps.

## Conclusion

The Kiswahili OIDP inventory had acceptable psychometrical properties among non-institutionalized adults 50 years and above in urban and rural areas of Tanzania. The impacts affecting their performances were relatively common but not very severe. Numerous dental problems contribute to the overall impact assessed among elderly Tanzanians in this study. To increase the applicability of the OIDP inventory in need assessment approaches and dental service planning, condition specific impacts should be assessed to support clinical measures of standard treatment needs.

## Competing interests

The author(s) declare that they have no competing interests.

## Authors' contributions

**IK**: Principal investigator, conceived of the study, designed the study, collected data, statistical analysis and manuscript writing

**ANÅ**: Main supervisor, designed study, statistical analysis, manuscript writing

**GS**: Participated in design of study and manuscript writing

**JM**: Participated in design of study, data collection and manuscript writing

**GT**: Have commented on the paper and provided valuable guidance for the OIDP scoring system

All authors read and approved the final manuscript

## Supplementary Material

Additional File 1 OIDP – Toleo la Kiswahili. The file provided is the Kiswahili version of the oral impacts on daily performances (OIDP) index. Click here for file
