# Supplementary material for: Psychometric properties and the prevalence, intensity and causes of oral impacts on daily performance (OIDP) in a population of older Tanzanians
Source: Health Qual Life Outcomes. 2006 Aug 27;4:56. doi: 10.1186/1477-7525-4-56 (PMC1579214; doi:10.1186/1477-7525-4-56)
Supplement: Additional File 1 — OIDP – Toleo la Kiswahili. The file provided is the Kiswahili version of the oral impacts on daily performances (OIDP) index. [file 1477-7525-4-56-S1.doc]

**OIDP - Toleo la KISWAHILI**

1) Katika kipindi cha miezi sita iliyopita ni mara ngapi umekuwa na tatizo katika mdomo wako na meno yaliyokuletea matatizo wakati wa **kula au kutafuna chakula**.

□ Sijapata tatizo

□ Chini ya mara moja kwa mwezi

□ Mara moja au mbili kwa mwezi

□ Mara moja au mbili kwa wiki

□ Mara 3-4 kwa wiki

□ Kila siku au karibu kila siku

2) Tatizo lako ni kubwa kiasi gani **unapokula au kutafuna chakula**?

□ Kubwa sana

□ Kubwa

□ Siyo kubwa

□ Siyo kubwa kabisa

3) Nini hasa tatizo lako la kinywa lililosababbisha upate matatizo wakati wa **kula au kutafuna chakula?**

| **Hali** | **Ndiyo** | **Hapana** |
| --- | --- | --- |
| ***Maumivu ya Jino*** |  |  |
| ***Jino linalolegea*** |  |  |
| ***Jipu la ufizi*** |  |  |
| ***Harufu mbaya*** |  |  |
| **Fizi zinazotoa damu** |  |  |

Jingine: (taja)____________________

4) Katika kipindi cha miezi sita iliyopita ni mara ngapi umekuwa na tatizo katika mdomo wako na meno yaliyokuletea matatizo katika **kuzungumza na kutamka maneno vizuri.**

□ Sijapata tatizo

□ Chini ya mara moja kwa mwezi

□ Mara moja au mbili kwa mwezi

□ Mara moja au mbili kwa wiki

□ Mara 3-4 kwa wiki

□ Kila siku au karibu kila siku

5) Tatizo lako ni kubwa kiasi gani la **kuzungumza na kutamka maneno vizuri**.

□ Kubwa sana

□ Kubwa

□ Siyo kubwa

□ Siyo kubwa kabisa

6) Kulikuwa na tatizo gani hasa la kinywa lililokufanya kupata taabu katika **kuzungumza na kutamka maneno vizuri**?

| **Hali** | **Ndiyo** | **Hapana** |
| --- | --- | --- |
| ***Maumivu ya Jino*** |  |  |
| ***Jino linalolegea*** |  |  |
| ***Jipu la ufizi*** |  |  |
| ***Harufu mbaya*** |  |  |
| **Fizi zinazotoa damu** |  |  |

Jingine, (taja)__________________

7) Katika kipindi cha miezi sita iliyopita ni mara ngapi umekuwa na tatizo katika mdomo wako na meno lililokufanya kupata taabu katika **kusafisha meno yako**?

□ Sijapata tatizo

□ Chini ya mara moja kwa mwezi

□ Mara moja au mbili kwa mwezi

□ Mara moja au mbili kwa wiki

□ Mara 3-4 kwa wiki

□ Kila siku au karibu kila siku

8) Tatizo lako ni kubwa kiasi gani **unaposafisha meno yako**?

□ Kubwa sana

□ Kubwa

□ Siyo kubwa

□ Siyo kubwa kabisa

9) Kulikuwa na tatizo gani hasa la kinywa lililokufanya kupata taabu katika **kusafisha meno yako**?

| **Hali** | **Ndiyo** | **Hapana** |
| --- | --- | --- |
| ***Maumivu ya Jino*** |  |  |
| ***Jino linalolegea*** |  |  |
| ***Jipu la ufizi*** |  |  |
| ***Harufu mbaya*** |  |  |
| **Fizi zinazotoa damu** |  |  |

Jingine, (taja)_____________

10) Katika kipindi cha miezi sita iliyopita ni mara ngapi umekuwa na tatizo katika mdomo wako na meno lililokufanya **kupata taabu katika kulala na kupumzika**?

□ Sijapata tatizo

□ Chini ya mara moja kwa mwezi

□ Mara moja au mbili kwa mwezi

□ Mara moja au mbili kwa wiki

□ Mara 3-4 kwa wiki

□ Kila siku au karibu kila siku

11) Tatizo lako ni kubwa kiasi gani **unapolala na kupumzika**?

□ Kubwa sana

□ Kubwa

□ Siyo kubwa

□ Siyo kubwa kabisa

12) Ulikuwa na tatizo gani la kinywa lililokupa taabu ya **kulala au kupumzika**?

| **Hali** | **Ndiyo** | **Hapana** |
| --- | --- | --- |
| ***Maumivu ya jino*** |  |  |
| ***Jino linalolegea*** |  |  |
| ***Jipu la ufizi*** |  |  |
| ***Harufu mbaya*** |  |  |
| **Fizi zinazotoa damu** |  |  |

Jingine, (taja)__________________

13) Katika kipindi cha miezi sita iliyopita ni mara ngapi umekuwa na tatizo katika mdomo wako na meno lililokufanya kupata taabu katika **kutabasamu, kucheka na kuonyesha meno yako bila kuona aibu**?

□ Sijapata tatizo

□ Chini ya mara moja kwa mwezi

□ Mara moja au mbili kwa mwezi

□ Mara moja au mbili kwa wiki

□ Mara 3-4 kwa wiki

□ Kila siku au karibu kila siku

14) Tatizo lako ni kubwa kiasi gani katika kutabasamu, **kucheka na kuonyesha meno bila kuona aibu**.

□ Kubwa sana

□ Kubwa

□ Siyo kubwa

□ Siyo kubwa kabisa

15) Ulikuwa na tatizo gani hasa la kinywa lililokupa taabu ya **kutabasamu, kucheka na kuonyesha meno bila kuoa aibu**?

| **Hali** | **Ndiyo** | **Hapana** |
| --- | --- | --- |
| ***Maumivu ya jino*** |  |  |
| ***Jino linalolegea*** |  |  |
| ***Jipu la ufizi*** |  |  |
| ***Harufu mbaya*** |  |  |
| **Fizi zinazotoa damu** |  |  |

Jingine, (taja)__________________

16) Katika kipindi cha miezi sita iliyopita ni mara ngapi umekuwa na tatizo katika mdomo wako na meno lililokufanya kupata taabu katika **kuwa katika hali yako ya kawaida (ya mhemko) bila kukereka.**

□ Sijapata tatizo

□ Chini ya mara moja kwa mwezi

□ Mara moja au mbili kwa mwezi

□ Mara moja au mbili kwa wiki

□ Mara 3-4 kwa wiki

□ Kila siku au karibu kila siku

17) Tatizo lako ni kubwa kiasi gani katika kuwa na **hali ya mhemko wa kawaida bila kukereka**

□ Kubwa sana

□ Kubwa

□ Siyo kubwa

□ Siyo kubwa kabisa

18) Ulikuwa na tatizo gani hasa la kinywa lililokupa taabu kuweza kuwa katika hali ya **kawaida ya mhemko bila kukereka**?

| **Hali** | **Ndiyo** | **Hapana** |
| --- | --- | --- |
| ***Maumivu ya jino*** |  |  |
| ***Jino linalolegea*** |  |  |
| ***Jipu la ufizi*** |  |  |
| ***Harufu mbaya*** |  |  |
| **Fizi zinazotoa damu** |  |  |

Jingine, (taja)__________________

19) Katika kipindi cha miezi sita iliyopita ni mara ngapi umekuwa na tatizo katika mdomo wako na meno linakupa taabu ya **kufanya kazi kubwa au majukumu ya kijamii**

□ Sijapata tatizo

□ Chini ya mara moja kwa mwezi

□ Mara moja au mbili kwa mwezi

□ Mara moja au mbili kwa wiki

□ Mara 3-4 kwa wiki

□ Kila siku au karibu kila siku

20) Tatizo lako ni kubwa kiasi gani katika **kufanya kazi kubwa na majukumu ya kijamii**

□ Kubwa sana

□ Kubwa

□ Siyo kubwa

□ Siyo kubwa kabisa

21 Ulikuwa na tatizo gani hasa la kinywa lililokupa taabu kuweza kufanya **kazi kubwa na majukumu ya kijamii**?

| **Hali** | **Ndiyo** | **Hapana** |
| --- | --- | --- |
| ***Maumivu ya jino*** |  |  |
| ***Jino linalolegea*** |  |  |
| ***Jipu la ufizi*** |  |  |
| ***Harufu mbaya*** |  |  |
| **Fizi zinazotoa damu** |  |  |

Jingine, (taja)__________________

22) Katika kipindi cha miezi sita iliyopita ni mara ngapi umekuwa na tatizo katika mdomo wako na meno lililokupa taabu katika **kufurahia pamoja na watu wengine**

□ Sijapata tatizo

□ Chini ya mara moja kwa mwezi

□ Mara moja au mbili kwa mwezi

□ Mara moja au mbili kwa wiki

□ Mara 3-4 kwa wiki

□ Kila siku au karibu kila siku

23) Tatizo lako ni kubwa kiasi gani katika **kufurahia pamoja na watu wengine**

□ Kubwa sana

□ Kubwa

□ Siyo kubwa

□ Siyo kubwa kabisa

***24) Ulikuwa na tatizo gani hasa la kinywa lililokupa taabu katika kuweza kufurahia pamoja na watu wengine.***

| **Hali** | **Ndiyo** | **Hapana** |
| --- | --- | --- |
| ***Maumivu ya jino*** |  |  |
| ***Jino linalolegea*** |  |  |
| ***Jipu la ufizi*** |  |  |
| ***Harufu mbaya*** |  |  |
| **Fizi zinazotoa damu** |  |  |

Jingine, (taja)__________________
